# Prodigious Fæcal Accumulations in the Rectum

**Published:** 1847-05

**Authors:** 


*Prodigious Fæcal Accumulations in the Rectum.—To the Editor of the Boston Medical and Surgical Journal.*

Dear Sir,—On the 17th of July, 1845, I was called to see F. C., a young lady aged about 15 years. Was informed that she had several times menstruated imperfectly, that she was somewhat troubled with costiveness, and that she had had no evacuation from the bowels during the three days last past. Tongue coated, white, not dry, skin hot and dry, pulse somewhat too frequent, and complained of pain in her head, with perfect loss of appetite. Prescription. Directed her to take six grains of the following pill mass every six hours. R. Soc. aloes, ʒ iij. ; g. scammony, ʒ iss. ; pulv. jalap, ʒ iij. 1-4 ; hyd. proto-cholo., ʒ i. ; sapo. cast., gr. xv. ; nit. pot., ʒ ss. ; tart. ant., ʒ j. ; ol. anise, arab. muc., aa q. s. to make a mass.

July 18.—Being about ten miles distant, I received a very urgent call to visit her ; found her in great pain, like the last pains of labor, the intermissions being very short, yet very perfect ; urgent and painful desire to pass urine, yet none had passed since the morning before (now 4 o'clock, P. M.). Cathartic pills have not operated, and was now informed that all the evacuations during the past two weeks had been but an occasional scanty discharge of mucus, and that such discharges were now being produced, the consequence of the excessive tenesmus. Deciding to introduce a catheter I attempted to pass a finger into the vagina, but was prevented by what appeared to be an unyielding mass, filling the whole pelvis, and pressing upward and forward so as to make it very difficult to pass the finger between it and the pubes. I accordingly carefully insinuated the point of a silver catheter into the urethra and passed it into the bladder, and discharged a quart or more of urine. The tenesmus still continued, and the acuteness of the pain was somewhat relieved, but the involuntary straining effort which characterizes the closing throes of labor still continued. With considerable difficulty I now passed a large-sized gumelastic catheter into the rectum, and through a mass of fæcal matter, some ten inches, when adapting a syringe to the external end of the tube, I succeeded by dint of persevearance in forcing warm water through the plugged orifice of the upper end. After sending up about a quart of fluid, the catheter was withdrawn, and in two or three minutes more than a gallon of fæcal matter followed, consisting almost entirely of the seeds of raspberries. After another small evacuation, which followed in a few minutes, she became entirely comfortable. The next day I was again called, and finding much the same symptoms, resorted to the same means, and obtained a similar result. After this the urinary bladder and the rectum evacuated themselves without aid, and raspberry seeds continued to appear in the fæces for several days longer, though none had been eaten during the week previous to my first callling on her. Since that time she has enjoyed her usual health.

I present this case to the notice of the profession, not on account of any peculiarity of the practice ; indeed I think it was but what was indicated, and would have readily suggested itself to any reflecting physician ; but 1. To show that a vast amount of fæcal matter may accumulate in the rectum, and also above the sigmoid flexure of the colon, while the sensibility of the mucous membrane remains low as in cases of constipation, but that when this sensibility is increased, as it was in this case by the cathartic, violent symptoms are the consequence ; and 2, That when the pelvis becomes sufficiently full to distend the perineum, the action of those muscles associated in the function of expelling the contents of the pelvic viscera is excited, and if this distention be proportionally increased their action becomes intermittent and involuntary. This phenomenon we have all so frequently witnessed in parturition, when the head of the child fully occupies the pelvis and rests on the perineum, that we find it difficult to view it as but a specific accompaniment of that series of phenomena, the aggregate of which constitutes labor. Indeed so strong did this influence operate upon my mind, in this case, that when preparing to introduce the catheter, notwithstanding the youth of my patient, and the character of the family being above suspicion, I could not divest myself of the feeling that, upon the finger entering the vagina, the head of a fœtus would present itself. S. A. COOK.

*Buskirk's Bridge, N. Y., Jan.* 4, 1847.

